# Authors' reply

**DOI:** 10.4103/0970-1591.42634

**Published:** 2008

**Authors:** Shriram Joshi

**Affiliations:** Department of Urology, Jaslok Hospital, Mumbai

Dear editor,

I read with interest the letter by Raveenthiran *et al*. I was surprised and disappointed by the letter and its tone. Upset because the author neither properly read nor understood the editorial and the two articles on pediatric urology training. [1–4] One by an eminent Pediatric surgeon of Mumbai and for some time now the Dean of a reputed Medical School and second by an equally known pediatric urologist whose training began as a pediatric general surgeon. The letter is defending “turf war” which Paddy Dewan is asking in his article to avoid. The editorial attempted to address a need in India to develop the specialty of pediatric urology and not to kill it as has been suggested by the letter. All three articles accept this issue.

To answer the question “who can be easily and effectively trained at the least expense of resources?” - a quick answer is “not trained at least expense” but rather the training should produce the best trained pediatric urologist. Both pediatric surgeons and urologists have to undergo three years of general surgical training in which about 15-20% is urology even now. In a pediatric surgical training program, this level of urology exposure goes to around 30-40%, I have been told whereas in a urological training program, it is 100% urology including pediatric urology, but may not be enough neonatal and fetal urology. It is with this deficit in mind that the editorial accepts that a urologist be trained for a year in pediatric surgery! Pediatric urology includes not only neonatal and fetal urology but grownup children as well. Since the number of urologists available all over India is more than pediatric surgeons, the care of grownup children with pediatric urology problems is well taken care of.

The letter addresses the growing population in India of geriatrics and the falling birth rate, but obviously he has not seen the latest birth rates and the ever-increasing young population. It is important not to spare resources in training those who are to look after children, who are the future of this country. If such an approach is undertaken you will end up with inferior pediatric urologists. I feel it is easier to train urologists in pediatric care during a fellowship in pediatric urology. To assimilate essentials of advanced urological techniques like neuro-urology, multitude of endourological procedures, surgery in renal failure including renal transplantation, surgeons who have had limited exposure to these techniques, may find it difficult or take much longer to acquire the skills It takes three full years of residency in urology to learn “how to think and predict the pathophysiology of this urological problem, and the solution to follow”; how would a pediatric surgeon who is doing only 40% urology learn all the intricacies in just three years? All the dedicated pediatric units in India and even some separate pediatric hospitals in private setups, do not have a dedicated pediatric urologist. Everybody does everything, jack of all and master of none! Getting urologists to treat urological problems can hardly be called suicidal. It is only following good clinical practice! I wonder why pediatric surgeons do not want to dabble into pediatric cardiac, orthopaedic, neurosurgery etc etc - if we go by their arguments.

Nowadays most healthcare in India is going down the American path as our Government seems intent on taking the insurance route rather than healthcare for all. Most good healthcare facilities in India are in private hands unlike Europe and Australia, where healthcare is controlled by the state. If anything, our healthcare model has more similarity with USA model than any other model. There are many more urologists in training in India than pediatric surgeons. There are more urologists qualifying each year than pediatric surgeons. If the pediatric surgeons concentrated on treating neonates and children with non-urological ailments, it would prevent general surgeons from doing pedicatric surgical work.

Yes, it is true that at the moment pure pediatric urology is not commercially viable. If the author is advocating a part-time pediatric surgeon/urologist, what is wrong in having a urologist with fellowship in pediatric urology doing urology/pediatric urology? I feel the latter model is much safer as basic grounding in pathophysiology of urinary ailments is good.

That brings me to the vexed problem of publications. If this is not following the US pattern then what is? “Publish or perish” is the western style of training. Does publishing articles in indexed journals by a few centers under the guidance of the same few senior authors equivalent to training in pediatric urology? I beg to differ, but then everyone is entitled to his opinion and I leave it to the readers of this journal to decide. Indian urologists who have many publications in many disciplines like transplant, oncology, BPH, GU Tuberculosis, Endourology, neuro-urology, andrology and have a healthy fifth of the articles in pediatric urology. It would be interesting if the author could produce names of institutions that do full justice to pediatric urology. Pediatric centers lack even the basic exposure to endourology, laparoscopy and urodynamics.

I can go on but what I would like to see is an end to this “Turf War”, establishment of many children's hospitals all over the country, with super-specialties in pediatric age groups including pediatric urology, practicing in a cordial, cooperative milieu and delivering the best care to the child. I will not see it in my lifetime, but I hope some of my younger colleagues will have the pleasure of working in such hospitals.
